# Coffee

**Published:** 1828-10

**Authors:** 


### II. Analytical.

14. *Coffee.* In the *third volume* of the *Dict. de Scien. Med.* there is an interesting article on Coffee, by a distinguished French physician, Dr. Nysten. The Arabs were the first who prepared a drink from the seeds of this plant. Its English and French names are derived from the Turkish name *Caouhe.* It is curious to trace the migration of this celebrated plant from the old to the new world. Near the end of the 16th century it was carried by the Dutch from Moka to Batavia, and soon afterwards from the latter place to Amsterdam; whence in 1713 M. Resson introduced it into the green houses of the royal garden, at Paris. From thence it was transported to the West Indies, by M. Declieux, who during his voyage deprived himself of a portion of his own drinking water, to refresh and sustain the young plant. It was first cultivated in the islands of Martinico, St. Domingo and Gaudeloupe.

Dr. Nysten treats of the qualities and effects of Coffee under two heads, *burnt* and *unburnt.* Its effects in the former state being well known, we shall pass over what he says concerning them, to present our readers with a translation of what he has collected relative to the medicinal powers of

*Coffee, unburnt.* "Coffee not roasted or torrefied imparts to water a greenish yellow colour; from whence is derived the name *lemon* coffee, which has been given to this drink, recommended by Andry (*Traite des alimens du careme*) and by Rostan and Ryhiner (*Acta Helvetica* tom. V. p. 384.) But it is, principally, at a later period, that the attention of physicians has been called, by professor Grindel, to unburnt Coffee; which he regards (*ouvr. periodiques cites*) as a good substitute for the Peruvian Bark. It was at the clinical establishment of the imperial University at Dorpat, in Russia, that this author made his experiments; and it was especially in intermittent fevers, that this medicine was administered to advantage. It was, however, given with success as a tonic in many other maladies. M. Grindel has given it in powder, in decoction, and in extract. To prepare the powder, the Coffee covered with water is to be exposed to a moderate fire, until it becomes moist; it is then to be placed in an oven, so temperately heated as not to burn it, and left to dry, when it may be easily pulverized. To make a strong decoction, the author directs us to boil an ounce of Coffee in 18 ounces of water, until it is reduced to six: he advises that we should prepare the extract in earthen, instead of iron vessels.

"Of more than 80 cases of intermittent fever he scarcely found one which resisted the action of Coffee, prepared in one of these modes. In powder, the strongest dose which he gave, was a scruple every two or three hours. It rarely happened that the most obstinate intermittents did not yield to two ounces of this powder. Sixteen ounces of the decoction were found to answer the same purpose. M. Grindel frequently gave the decoction to second the action of the powder. The dose of the extract is variable and similar to that of the extract of cinchona. When we administer Coffee as a tonic, the extract has appeared to M. Grindel to be the best preparation. Among many interesting cases in which this medicine was administered successfully as a tonic, we find that of a man, upwards of thirty years of age, who having been cured of a dropsy of the chest, remained in a state of emaciation accompanied with diarrhœa, which resisted the action of the Bark. After having laid aside for some time every kind of medicine, and finding that the disease still continued, recourse was had to the extract of Coffee, combined with a little opium, and dissolved in water; but the mixture occasioned pain in the bowels, and they afterwards limited themselves to the Coffee alone, in the dose of two spoonfuls a day, dissolved in about six ounces of water. This medicine gradually moderated the disease and improved the condition of the patient, so that when he had taken about twelve ounces at the end of two months, he was quite cured.

"A woman had lost, in a few years, nine children, who had died between the ages of two and three years, of atrophy attended, in the close, with colliquative diarrhœa. The tenth, aged eighteen months, was on the point of falling under the same disease. The Bark passed unaltered from the bowels, and other medicines were given without effect, when they resorted to the decoction of Coffee, with a little tragacanth and extract of tormentil. Afterwards, however, they limited themselves to the decoction of Coffee, which was continued for nine weeks, when the child was perfectly cured.

"The facts which we have cited from Dr. Grindel, and which might be collected from the *Bibliotheque Medicale* are sufficient to establish the efficacy of unburnt Coffee, as an excellent tonic and febrifuge."

